# Ferrostatin-1 alleviates experimental cerebral malaria by regulating immune cell functions and brain endothelial ferroptosis

**DOI:** 10.1016/j.ijpddr.2025.100630

**Published:** 2025-12-17

**Authors:** Shijie Yao, Xiaoliang Zhou, Ting Liao, Chao Yao, Mengna Sun, Haojie Gou, Xueyuan Hu, Junyu Liu, Li Zheng, Yan Zhao, Yaming Cao

**Affiliations:** a Department of Immunology, College of Basic Medical Sciences, China Medical University, Shenyang, Liaoning, China; b National Clinical Research Center for Laboratory Medicine, Department of Laboratory Medicine, The First Hospital of China Medical University, Shenyang, Liaoning, China

**Keywords:** *Plasmodium berghei*, Experimental cerebral malaria, Ferroptosis, Ferrostatin-1, Pro-inflammatory reaction, Brain microvascular endothelial cells

## Abstract

Cerebral malaria (CM), a life-threatening complication of *Plasmodium falciparum* infection, is characterized by dysregulated immune responses and blood-brain barrier (BBB) damage. In this study, we found that iron metabolic disorders occurred in the spleen and brain tissues in response to *Plasmodium berghei* ANKA (PbA) infection in a murine CM model. PbA infection promoted lipid peroxidation and induced ferroptosis, manifested as the accumulation of iron ion, elevation of reactive oxygen species and lipid peroxide, upregulated expression of the ferroptosis-related protein TFRC and ACSL4, and downregulated expression of SLC7A11 and GPX4. Ferrostatin-1 (Fer-1), is widely used as a reference compound as a synthetic radical-trapping antioxidant, which inhibits ferroptosis by suppressing lipid peroxide formation. Intervention with Fer-1 ameliorated iron metabolic disorders, reduced lipid peroxidation, decreased parasitemia, extended survival time, alleviated neurological symptoms, and improved BBB integrity. Mechanistically, Fer-1 exerted dual-axis regulation: firstly, enhancing the antigen-presenting capacity of dendritic cells (DCs) by upregulating MHC II, CD80/86, promoting M1 polarization of macrophages, modulating CD4^+^ T cell responses to increase IFN-γ^+^ Th1 cells and Treg cell proportions for balancing pro-inflammatory and anti-inflammatory reactions; secondly inhibiting ferroptosis in brain microvascular endothelial cells, downregulating chemokines CXCL9/CXCL10 and adhesion molecules ICAM-1/VCAM-1, and reducing cerebral infiltration of CD8^+^ T cells. Our study confirms that Fer-1 alleviates ECM pathological progression through dual mechanisms "immune activation-endothelial protection", providing a novel ferroptosis-targeted strategy for CM prevention and treatment.

## Highlights

• *Plasmodium* *berghei* ANKA infection triggers ferroptosis in the spleen and brain of an experimental cerebral malaria model.
• Ferrostatin-1 prevents cerebral malaria by reducing parasitemia and preserving blood-brain barrier integrity.
• Ferrostatin-1 functions through a dual mechanism by modulating peripheral immune cells and protecting the brain endothelium.

## Graphical abstract

**Figure:**
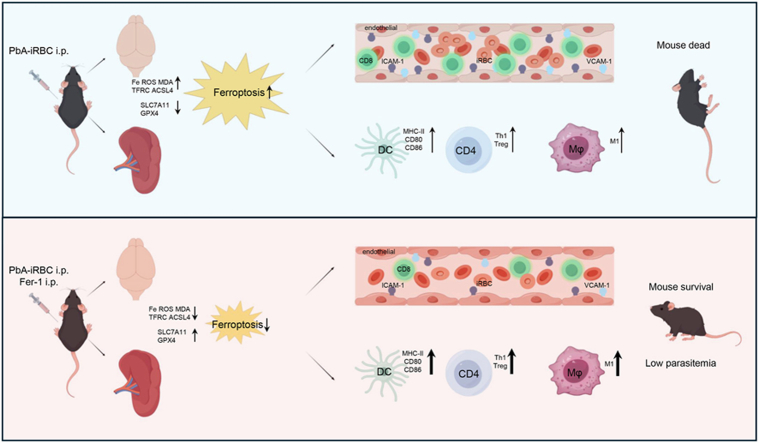

## 1 Introduction

Cerebral malaria (CM), the most life-threatening complication of *Plasmodium falciparum* infection, has a mortality rate of up to 25 %. Even with timely intervention, survivors often suffer from acute neurological deficits or persistent cognitive impairments (de Lima et al., 2025). Its core pathological features include disruption of the blood-brain barrier (BBB) integrity, inflammatory cell infiltration in the brain parenchyma, and neuronal damage (Song et al., 2022). Clinical and basic studies have shown that sequestration of infected red blood cells (iRBC) in cerebral microvessels, imbalance of the pro-inflammatory/anti-inflammatory cytokine network, and endothelial dysfunction are key pathological links leading to coma in severe cases (Hunt et al., 2014). Currently, the experimental cerebral malaria (ECM) model established by infecting C57BL/6 mice with *Plasmodium berghei* ANKA (PbA) has become a classic tool for studying the pathological mechanisms of CM (Albrecht-Schgoer et al., 2022). This model recapitulates typical brain pathological changes of human CM, including increased BBB permeability, focal cerebral hemorrhage, and brain edema, providing a reliable research vehicle for dissecting the cascade of neuroinflammation (Swanson et al., 2016).

Iron, an essential nutrient for *Plasmodium* growth, plays a dual role in CM pathogenesis: host iron deficiency inhibits parasite proliferation, while iron overload exacerbates oxidative stress and inflammatory damage (Clark et al., 2014). Fe^2+^ released by *Plasmodium*-mediated hemoglobin degradation catalyzes Fenton reactions, inducing accumulation of lipid peroxidation products—a process highly consistent with the core features of ferroptosis (Percario et al., 2012). As an iron-dependent non-apoptotic cell death triggered by impaired lipid peroxide clearance, ferroptosis has been confirmed to regulate cellular functions in tumors and neurodegenerative diseases (Jiang et al., 2021). In the context of malaria, ferroptosis is implicated in pathogenesis, including inducing biochemical changes in *Plasmodium* and reducing hepatic-stage parasite viability (Kain et al., 2020; Sena-Dos-Santos et al., 2021). Moreover, the antimalarial efficacy of dihydroartemisinin, a first-line antimalarial compound, is thought to be associated with its mediation of ferroptosis (Du et al., 2019; Li et al., 2022).

Ferrostatin-1 (Fer-1), one of the earliest reported ferroptosis blockers, is widely used as a reference compound as a synthetic radical-trapping antioxidant, which inhibits ferroptosis by suppressing lipid peroxide formation (Scarpellini et al., 2023). This inhibitor has been confirmed to suppress cell death in models of Huntington's disease (HD), periventricular leukomalacia (PVL), renal dysfunction, and breast cancer (Skouta et al., 2014; Chen et al., 2017). In ECM, Fer-1 has been shown to protect neurons by inhibiting GPX4-dependent lipid peroxidation (Liang et al., 2022). However, direct experimental evidence regarding its regulation of immune cell responses and brain endothelial cell integrity during ECM remains lacking. Therefore, in our study, we systematically explored the regulatory mechanisms of Fer-1 in the "immune activation-vascular protection" dual axis to provide new theoretical foundations for targeted CM therapy.

## 2 Materials and methods

### 2.1 Mice and infection

6-8-week-old female C57BL/6 mice were purchased from Beijing Huafukang Biotechnology Co., Ltd. All mice were maintained with the same feeding conditions and a SPF environment within a treatment room (12-h light/dark cycle, 22 ± 2 °C, 60–65 % relative humidity) and housed for at least one week before infection. Animal experiments were performed in accordance with the approved protocols (CMU2023112) by the Institutional Animal Care and Use Committee of Laboratory Animals of China Medical University.

Infections were initiated by intraperitoneal (i.p.) injection with 1 × 10^6^ *Plasmodium berghei* ANKA (PbA) - iRBC. Ferroptosis inhibitor (Ferrostatin-1, Fer-1) was purchased from Sigma-Aldrich, USA. Fer-1 administrations were injected via i. p., on days 0, 2, and 4 post-infections (5 mg/kg). Mice were placed within three groups: Normal (uninfected+3 % DMSO treated), PbA (infected+3 % DMSO treated), PbA + Fer-1 (infected + Fer-1 treated). From 3 days post-infection (dpi), tail vein blood was collected every other day from mice in the PbA (n = 7) and PbA + Fer-1 groups (n = 8) to prepare blood smears, which were stained with Giemsa for microscopic quantification, while the survival status of the mice was monitored daily. Neurological symptoms were evaluated and recorded using the ECM clinical grading scale, specifically defined as stage 0 (asymptomatic), stage 1 (mild piloerection), stage 2 (moderate piloerection with mild ataxia), stage 3 (ataxia and paralysis), and stage 4 (convulsions and coma) (Linares et al., 2013).

### 2.2 Detection of tissue MDA and tissue/serum iron levels

On 5 dpi, mice in each group were anesthetized and euthanized by cervical dislocation. Approximately 0.1 g of spleen/brain tissue was added to 1 mL of extraction solution, homogenized in an ice bath, centrifuged at 8000 g for 10 min (min) at 4 °C, and the supernatant was collected. The levels of MDA and total iron in spleen and brain tissues homogenates were determined based on the protocols of the MDA assay kit (Beyotime, Shanghai, China) and total iron assay kit (Nanjing Jiancheng Bioengineering Institute, Nanjing, China). In addition, venous blood samples were collected, allowed to stand at room temperature (RT) for 4 h (h), and subsequently centrifuged at 10,000 rpm for 10 min to separate the serum. The serum Fe^2+^ concentration was then determined according to the protocol provided with commercial detection kits (Nanjing Jiancheng Bioengineering Institute, Nanjing, China).

### 2.3 Preparation of spleen and brain single-cell suspensions

On day 5 post-infection, mice were euthanized by cervical dislocation. The spleen was immediately harvested and placed in a sterile Petri dish containing pre-chilled PBS. The tissue was gently homogenized using a sterile grinding rod until complete dispersion was achieved. The resulting suspension was filtered through a 70 μm cell strainer to remove tissue debris, and RBC lysis buffer (Beijing Solarbio Science & Technology Co., Ltd.) was added to the filtrate. After incubation at room temperature for 5 min, the sample was washed three times with pre-chilled PBS via centrifugation (1600 rpm, 4 °C, 6 min per cycle). The final cell pellet was resuspended in pre-chilled PBS and adjusted to a concentration of 1 × 10^7^ cells/mL for downstream applications. Concurrently, the brain was rapidly excised and transferred to a sterile Petri dish containing pre-chilled PBS. It was homogenized using a sterile tissue grinder to prepare a uniform suspension. This homogenate was carefully layered onto a centrifuge tube containing a 30 % Percoll solution, ensuring minimal disturbance to maintain a distinct interface. Following centrifugation at 2000 rpm for 20 min, the upper layer containing myelin debris was aspirated without disturbing the underlying cell layer. The lower-layer cells were collected and washed twice with pre-chilled PBS. Subsequently, 3 mL of RBC lysis buffer was added, and the suspension was incubated on ice for 5 min to eliminate residual RBCs. Finally, the cells were washed additional times with pre-chilled PBS, counted using a hemocytometer, and resuspended at an appropriate concentration for subsequent experimental procedures.

### 2.4 Detection of ROS and lipid peroxidation levels

Spleen and brain cell suspensions were washed with 1 mL PBS, and the supernatant was discarded. The probes DCFH-DA (Beyotime, China), C11 BODIPY581/591 (Thermo Fisher Scientific, USA), and Liperfluo-1 (MCE, USA) were added respectively, followed by incubation at 37 °C in the dark for 30 min. The cells were then washed three times with 1 mL PBS, and after discarding the supernatant, the cell pellets were resuspended in 200 μL PBS for FACS Celesta (BD Biosciences, USA). The relative level of C11 BODIPY581/591-labeled lipid peroxidation was calculated via Green Fluorescence (510 nm)/Red Fluorescence (591 nm).

### 2.5 BBB integrity detection

After mice developed ECM symptoms, 200 μL of 2 % Evans blue (EB) dye was injected via tail vein. One hour later, mice were anesthetized, and the abdominal cavity was opened for cardiac perfusion with PBS until clear effluent was observed. The brain was harvested, photographed, placed in a 4 mL EP tube, added with 1 mL formamide, incubated at 37 °C for 48 h, centrifuged at 3000 rpm for 15 min, and 100 μL supernatant of each sample was aspirated into 96-well plates for OD value detection at 630 nm using a microplate reader (SpectraMax ABS plus, USA).

### 2.6 Flow cytometry

For cell membrane surface staining, Fcγ receptors were first blocked with anti-CD16/CD32, then surface fluorescent antibodies were added and incubated at 4 °C in the dark for 30 min, followed by centrifugation at 1600 rpm for 6 min at 4 °C for washing. Before intracellular cytokine staining, cells were incubated with 200 μL of RPMI 1640 medium containing 50 ng/mL PMA, 10 μg/mL BFA and 1 μg/mL Ionomycin (Shandong Sparkjade Biotechnology Co.,Ltd) in a 37 °C, 5 % CO_2_ environment for 4 h. After completing surface staining, cells were treated with Foxp3/Transcription Factor Staining Buffer Set (eBioscience, USA) at 4 °C in the dark for 40 min, and then antibodies were added for incubation. Finally, all samples were resuspended in 200 μL flow cytometry buffer for detection by FACS Celesta and analyzed with FlowJo v10 software (Tree Star). The antibodies were used including BV605-CD11c (N418, Biolegend), APC-MHC-II (M5/114.15.12, eBioscience), BV421-CD86 (GL1, Biolegend), Percp-Cy5.5-CD80 (16-10A1, Biolegend), APC-Cy7-CD45 (30-F11, Biolegend), Percp-CD4 (GK1.5, Biolegend), BV510-CD8 (53–6.7, Biolegend), PE-Cy7-T-bet (4B10, Biolegend), APC-IFN-γ (XMG1.2, Biolegend), e450-TNF-α (MP6-XT22, eBioscience), FITC-CD4 (GK1.5, Biolegend), Percp-Cy5.5-CD25 (PC61, BD), APC-Foxp3 (FJK-16s, eBioscience), Percp-Cy5.5-F4/80 (BM8, Biolegend), FITC-CD11b (M1/70, Biolegend), APC-CD206 (C068C2, Biolegend), PE-Cy7-CD31 (390, eBioscience), APC-VCAM-1 (429, eBioscience), FITC-ICAM-1 (KAT-1, eBioscience).

### 2.7 Enzyme-linked immunosorbent assay (ELISA)

The prepared spleen single-cell suspension, adjusted to a concentration of 1 × 10^7^ cells/mL, was seeded in duplicate into 24-well culture plates at 0.5 mL per well and incubated at 37 °C under a humidified atmosphere containing 5 % CO_2_ for 48 h. The culture supernatants were subsequently collected for ELISA analysis. Commercially available ELISA kits (R&D Systems, USA) were used to quantify the levels of IFN-γ, IL-6, IL-10, and TNF-α in the splenocyte culture supernatants at 5 dpi. One day in advance, 100 μL of coating solution was added to each well of a 96-well plate and coated overnight at 4 °C. The coating solution was discarded, and the plate was washed 3 times with PBST. Then, 200 μL of 1 % BSA was added to each well for blocking at 37 °C for 1 h, followed by 3 washes with PBST. Next, 100 μL of test samples and gradient standards were added to the corresponding wells in duplicate, incubated at 37 °C for 2 h, and washed 3 times with PBST, with the plate strips thoroughly patted dry on filter paper. Enzyme-labeled secondary antibodies (100 μL/well) were added and incubated at 37 °C for 1 h, followed by 7–8 washes with PBST and thorough drying on filter paper. Substrate working solution (100 μL/well) was added and allowed to develop in the dark for 10–15 min, then stop solution (50 μL/well) was added to terminate the reaction. The OD values were measured at 450 nm using a microplate reader (SpectraMax ABS plus, USA), and the cytokine concentrations in the samples were calculated according to the standard curve formula.

### 2.8 Purification of iRBCs

Peripheral blood of mice was collected aseptically with anticoagulation, and 1 mL of blood was added to 5 mL of PBS buffer for mixing. The mixture was slowly layered over 5 mL of 60 % Nycodenz solution (Alere Technologies AS, Norway). After clear layering, centrifugation was performed at 2500 rpm (himac CT6E, HITACHI, Japan) for 25 min at RT. Following centrifugation, trophozoites at the interface of the PBS buffer and Nycodenz solution were aspirated with a sterile glass tube and washed 2–3 times with PBS, while uninfected RBCs and ring stage iRBCs were pelleted. An appropriate number of cells was smeared, fixed with methanol and air-dried, stained with Giemsa solution at RT for 15 min, and the purity of iRBCs was identified under a microscope, which was usually greater than 95 %.

### 2.9 iRBCs stimulate bEnd.3 cells

bEnd.3 cells (Qinqi, Shanghai) were seeded at a density of 1 × 10^5^ cells per well in 24-well plates. After 24 h of culture for cell adhesion, the cells were pretreated with 2 μM Fer-1 for 4 h, followed by the addition of 1 × 10^6^ iRBCs per well. The co-culture was incubated in a 37 °C, 5 % CO_2_ incubator for 12 h. Upon completion of stimulation, RBC lysis buffer was added to remove residual RBCs, and bEnd.3 cells were collected for subsequent experimental detection.

### 2.10 Mitochondrial membrane potential (MMP) assay

The MMP of bEnd.3 cells was assessed using the TMRE assay kit (Beyotime, C2001S). Briefly, 2 × 10^5^ bEnd.3 cells per group were incubated with TMRE staining solution at 37 °C for 30 min in the dark. The cells were then gently washed twice with pre-warmed PBS to remove excess and non-specifically bound dye. Following centrifugation and removal of the supernatant, the cell pellets were resuspended in 200 μL of pre-warmed PBS, and fluorescence intensity was immediately measured using a BD FACS Celesta.

### 2.11 Western blotting

Spleen cells or collected bEnd.3 cells were centrifuged at 600 g for 10 min at 4 °C, and the supernatant was discarded. The pellets were treated with RIPA lysis buffer (Sangon Biotech, Shanghai, China) containing phosphatase inhibitors on ice, sonicated for disruption, and then centrifuged at 12,000 rpm for 15 min at 4 °C. The supernatants were aliquoted and stored at −80 °C. Protein concentration was determined by BCA assay. After adding loading buffer, samples were heated at 100 °C for 10 min to denature proteins. Proteins were separated by SDS-PAGE and transferred to PVDF membranes, which were blocked at RT for 1 h and incubated with primary antibodies against TFRC, ACSL4, SLC7A11, GPX4 (ABclonal, China), and β-actin (ZSGB-BIO, China) at 4 °C overnight. The next day, membranes were washed three times with TBST, incubated with diluted horseradish peroxidase (HRP)- conjugated secondary antibodies at RT for 1 h (ZSGB-BIO, China) was added, washed again with TBST, and developed by ECL. Images were captured using a Tanon imaging system, and gray values were analyzed by ImageJ for statistical analysis.

### 2.12 Real-time quantitative PCR analysis

Fifty to 100 mg of mouse brain tissue was taken and added to Trizol (Cowin Biotech, China) for total RNA extraction. After removing genomic DNA from 1 μg of RNA, cDNA was synthesized by reverse transcription (Takara, Japan). Using the designed specific primers for *Cxcl9*, *Cxcl10* and the internal reference *β-actin* (*β-actin* F: 5′-GATTACTGCTCTGGCTCCTAGC-3′, R: 5′-GACTCATCGTACTCCTGCTTGC-3’; *Cxcl9* F: 5′-CCGAGGCACGATCCACTACA-3′, R: 5′-AGTCCGGATCTAGGCAGGTTTG-3’; *Cxcl10* F: 5′-GCCGTCATTTTCTGCCTCAT-3′, R: 5′-GCTTCCCTATGGCCCTCATT-3′), a 20 μL reaction system was prepared (containing SYBR Green Master Mix, primers and cDNA template). qPCR was performed through pre-denaturation, 40 amplification cycles and melting curve analysis. After obtaining the Ct values, the *2*^*-ΔΔCt*^ method was used to calculate the relative expression levels of the target genes.

### 2.13 Statistical analysis

Statistical analyses were performed using GraphPad Prism 10.1.2 software. All data were presented as mean ± standard deviation (Mean ± SD). One-way analysis of variance (ANOVA) and Student's *t*-test were used to compare the significance of differences in means among groups, while survival analysis was conducted via the Log-rank test. A *P*-value <0.05 was considered statistically significant.

## 3 Results

### 3.1 PbA infection triggers iron metabolic disorders and lipid peroxidation-driven ferroptosis in mouse spleen

**Fig. 1:**
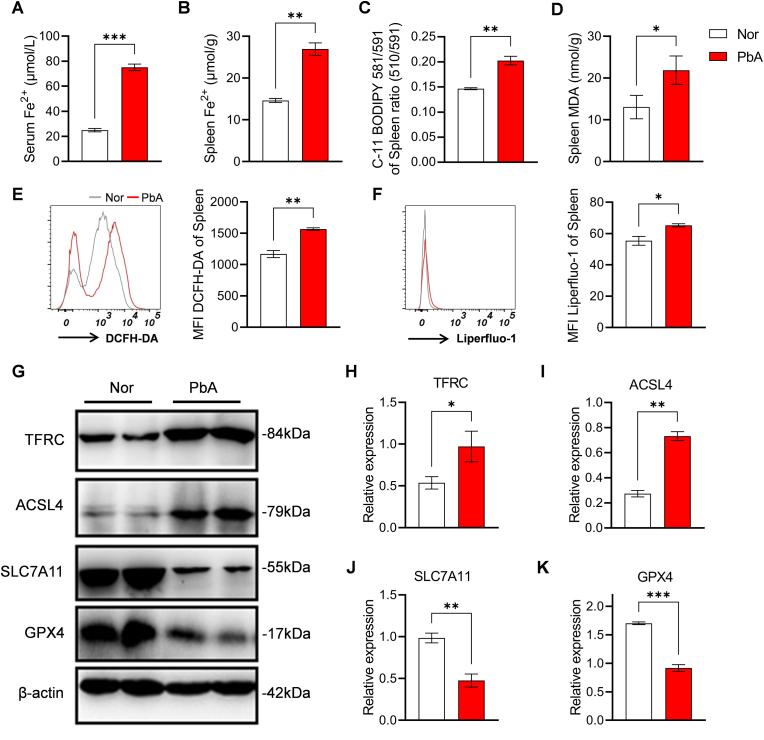
**Effects of PbA infection on iron metabolism, oxidative stress, and ferroptosis-related indicators in mouse spleen**. **A.** Concentration of serum Fe^2+^. **B.** Total iron content in spleen tissue. **C.** Lipid peroxidation level detected by C11-BODIPY 581/591 (MFI) based on fluorescence quenching effect, and was calculated as the ratio of green fluorescence (510 nm) to red fluorescence (591 nm). **D.** Content of MDA in spleen. **E.** Flow cytometry histogram and quantitative analysis (MFI) of ROS labeled by DCFH-DA. **F.** Flow cytometric detection results of lipid peroxidation specifically labeled by Liperfluo-1 (histogram and MFI). **G.** Western blot analysis of key ferroptosis-regulating proteins (TFRC, ACSL4, SLC7A11, GPX4) with β-actin as the internal reference. **H-K.** Statistical analysis of relative expression level of TFRC, ACSL4, SLC7A11 and GPX4 protein. Data are presented as mean ± SD. Inter-group differences were analyzed by Student's *t*-test, with *p < 0.05, **p < 0.01, and ***p < 0.001 considered statistically significant.

To investigate the association between *Plasmodium* infection and ferroptosis, we first measured ferroptosis-related parameters in mice infected with PbA. Results showed that serum Fe^2+^ and splenic Fe^2+^ levels significantly increased on 5 dpi (Fig. 1A and B), indicating iron metabolic disorders and iron ion accumulation during PbA infection. Concomitantly, splenic lipid peroxidation levels labeled by C11BODIPY581/591 increased (Fig. 1C), malondialdehyde (MDA) content rose (Fig. 1D), and fluorescence intensities of reactive oxygen species (ROS) labeled by DCFH-DA (Fig. 1E) and lipid peroxides labeled by Lipidperoxid-1 (Fig. 1F) were enhanced. At the protein level, expressions of transferrin receptor (TFRC) and acyl-CoA synthetase long-chain family member 4 (ACSL4) in the spleen of infected mice were upregulated (Fig. 1G–I), while expressions of key ferroptosis inhibitory proteins solute carrier family 7 member 11 (SLC7A11) and glutathione peroxidase 4 (GPX4) were significantly downregulated (Fig. 1G–J-K). These results indicate that PbA infection induces ferroptosis in mouse splenocytes through iron metabolic imbalance and lipid peroxidation.

### 3.2 Fer-1 intervention ameliorates iron metabolic disorders and lipid peroxidation in PbA-infected mice

**Fig. 2:**
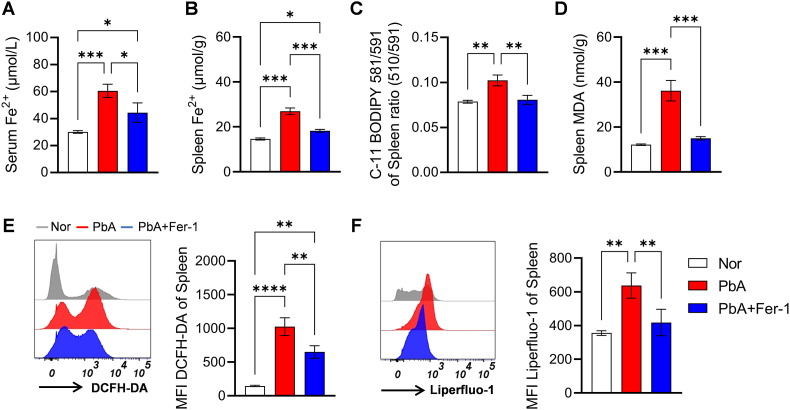
**Intervention effects of Fer-1 on spleen iron metabolism and oxidative stress indicators in PbA-infected mice**. **A.** Concentration of serum Fe^2+^. **B.** Total iron content in spleen tissue. **C.** Lipid peroxidation level detected by C11-BODIPY 581/591 (MFI) based on fluorescence quenching effect, and was calculated as the ratio of green fluorescence (510 nm) to red fluorescence (591 nm). **D.** Content of MDA in spleen. **E.** Flow cytometry histogram and quantitative analysis (MFI) of ROS labeled by DCFH-DA. **F.** Flow cytometric detection results of lipid peroxidation specifically labeled by Liperfluo-1 (histogram and MFI). Data are presented as mean ± SD. Inter-group differences were analyzed by One-way ANOVA, *p < 0.05, **p < 0.01, ***p < 0.001 and ****p < 0.0001 considered statistically significant.

Given that splenocytes of PbA-infected mice undergo ferroptosis, we further explored the interventional effects of the ferroptosis inhibitor Fer-1 by measuring serum and splenic iron metabolism and lipid peroxidation-related parameters on 5 dpi. Results showed that compared with PbA group mice, Fer-1 treatment reduced serum Fe^2+^ levels (Fig. 2A) and splenic Fe^2+^ content (Fig. 2B), decreased splenic lipid peroxidation levels labeled by C11BODIPY581/591 (Fig. 2C) and MDA content (Fig. 2D). Flow cytometry assays using DCFH-DA for ROS labeling (Fig. 2E) and Lipidperoxid-1 for lipid peroxide labeling (Fig. 2F) further revealed that Fer-1 treatment significantly downregulated splenic ROS and lipid peroxide levels. These findings indicate that Fer-1 effectively ameliorates iron metabolic disorders and alleviates lipid peroxidation induced by PbA infection.

### 3.3 Effects of Fer-1 intervention on disease progression and blood-brain barrier in PbA-infected mice

**Fig. 3:**
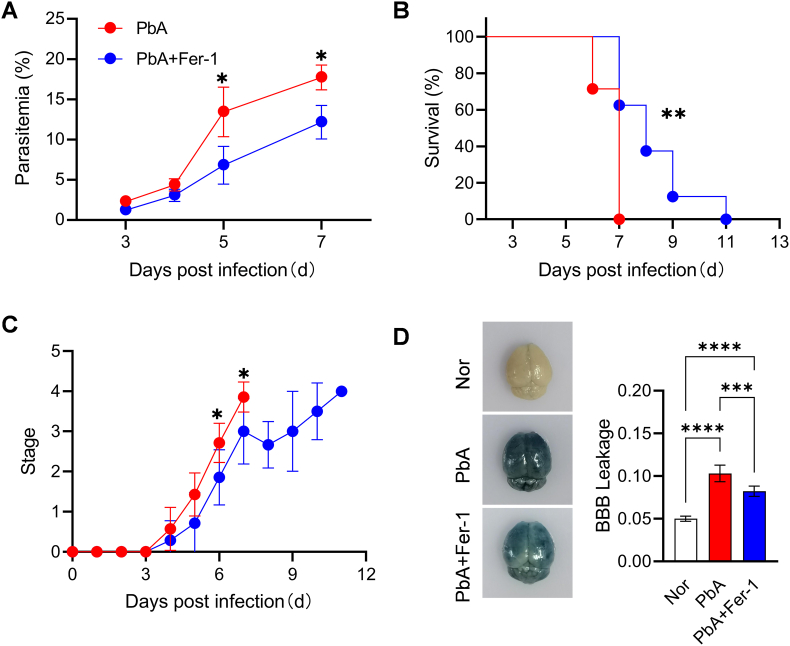
**Effects of Fer-1 on parasitemia, survival, disease progression, and BBB leakage in PbA-infected mice**. **A.** Statistics of parasitemia percentage at different days post-infection. **B.** Statistics of mouse survival percentage at different days post-infection. **C.** Clinical grading of neurological symptoms in mice. **D.** Detection of brain tissue appearance and BBB leakage levels in each group of mice. Data are presented as mean ± SD. Survival analysis was performed using the Log-rank (Mantel-Cox) test. Inter-group differences were analyzed by Student's t-test or One-way ANOVA, *p < 0.05, **p < 0.01, ***p < 0.001 and ****p < 0.0001 considered statistically significant.

To clarify the impact of Fer-1 on the course of ECM, we dynamically monitored parasitemia, survival, disease progression, and evaluated BBB integrity in infected mice. Results showed that parasitemia levels in PbA + Fer-1 group mice were significantly lower than those in the PbA group on 5 and 7 dpi (Fig. 3A), indicating that intervention of Fer-1 can inhibit parasite proliferation to a certain extent. Survival rate results showed that the PbA group first exhibited mortality on 6 dpi, accompanied by severe ECM-related neurological symptoms such as convulsions, paralysis, coma, and ataxia, with all mice dying by 7 dpi. Mice in the PbA + Fer-1 group also developed ECM symptoms on 6 dpi, but with milder symptoms and a slower mortality progression, dying entirely on 11 dpi, demonstrating prolonged survival time (Fig. 3B). Clinical grading of ECM neurological symptoms showed that the PbA group exhibited ataxia, paralysis, coma, and other symptoms earlier and more severely, with significant statistical differences on 6 and 7 dpi (Fig. 3C). BBB leakage detection revealed reduced BBB leakage in the PbA + Fer-1 group compared to the PbA group (Fig. 3D), indicating that Fer-1 alleviates BBB damage caused by *Plasmodium* infection and protects against neuropathological progression. These results demonstrate that correcting iron homeostasis imbalance significantly reduces parasitemia, prolongs survival time, alleviates ECM-related neurological symptoms, protects BBB integrity, and improves survival prognosis in CM mice.

### 3.4 Fer-1 regulates the activation and antigen-presenting function of dendritic cells (DCs)

**Fig. 4:**
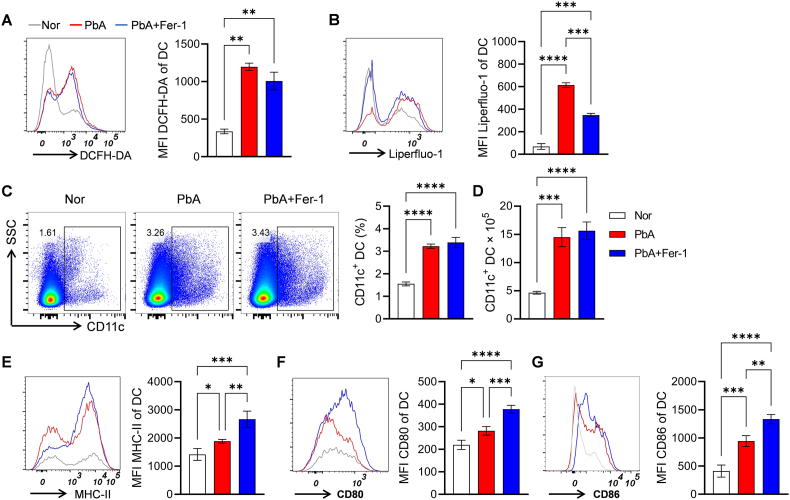
**Effects of Fer-1 on oxidative stress and phenotype of DCs in PbA-infected mice**. **A.** Flow cytometry histograms and quantitative analysis MFI of ROS in DCs labeled by DCFH-DA. **B.** Flow cytometry histograms and quantitative analysis MFI of lipid peroxidation in DCs labeled by Liperfluo-1. **C.** Flow cytometry scatter plots and statistical bar charts of the proportion of CD11c^+^ DCs in spleen. **D.** Statistical bar charts of the absolute cell counts of CD11c^+^ DCs in spleen. **E.** Flow cytometry histograms and MFI statistics of MHC-II on the surface of DCs. **F.** Flow cytometry histograms and MFI statistics of CD80 on the surface of DCs. **G.** Flow cytometry histograms and MFI statistics of CD86 on the surface of DCs. Data are presented as mean ± SD. Inter-group differences were analyzed by One-way ANOVA, *p < 0.05, **p < 0.01, ***p < 0.001 and ****p < 0.0001 considered statistically significant.

As the "initiator" of immune responses, DCs' innate recognition of *Plasmodium* is crucial for adaptive immune responses (Osii et al., 2020). Our results showed that after PbA infection, the levels of DCFH-DA-labeled ROS and Liperfluo-1-labeled lipid peroxides in DCs increased, indicating enhanced oxidative stress, while Fer-1 intervention reduced these levels (Fig. 4A–B). Flow cytometry analysis showed that the proportion of CD11c^+^ DCs increased in the PbA group, and Fer-1 intervention did not affect this proportional change and absolute cell counts (Fig. 4C and D). Further detection of DC surface molecules related to antigen presentation revealed that expressions of MHC-II, CD80, and CD86 in DCs of the PbA group were upregulated, and Fer-1 treatment further promoted the expressions of these molecules (Fig. 4E–G). These results suggest that Fer-1 promotes DC activation and antigen-presenting function by inhibiting oxidative stress in DCs.

### 3.5 Fer-1 regulates CD4^+^ T cell responses and functional polarization

**Fig. 5:**
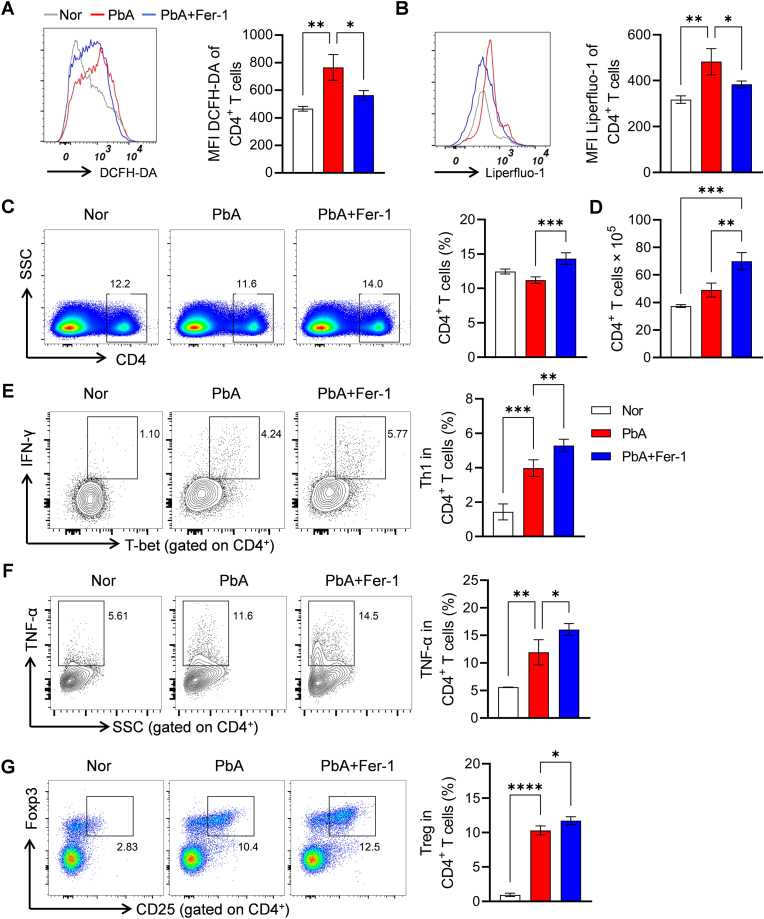
**Effects of Fer-1 on oxidative stress, proportion, and functional subsets of CD4^+^ T cells in PbA-infected mice**. **A.** Flow cytometry histograms and quantitative analysis MFI of ROS in CD4^+^ T cells labeled by DCFH-DA. **B.** Flow cytometry histograms and quantitative analysis MFI of lipid peroxidation in CD4^+^ T cells labeled by Liperfluo-1. **C.** Flow cytometry scatter plots and statistical bar charts of the proportion of CD4^+^ T cells in spleen. **D.** Statistical bar charts of the absolute cell counts of CD4^+^ T cells in spleen. **E.** Flow cytometry scatter plots and proportion of Th1 cells (IFN-γ^+^T-bet^+^) in CD4^+^ T cells. **F.** Flow cytometry scatter plots and proportion of TNF-α^+^ in CD4^+^ T cells. **G.** Flow cytometry scatter plots and proportion of Treg cells (CD25^+^Foxp3^+^) in CD4^+^ T cells. Data are presented as mean ± SD. Inter-group differences were analyzed by One-way ANOVA, *p < 0.05, **p < 0.01, ***p < 0.001 and ****p < 0.0001 considered statistically significant.

CD4^+^ T cells have been demonstrated to play a central role in the immune control of *Plasmodium* infection, where IFN-γ- and TNF-α-producing Th1 cells are critical for controlling blood-stage malaria, while Treg cell-mediated immunomodulatory responses help limit immune-mediated host damage (Kurup et al., 2019). Our results showed that PbA infection increased DCFH-DA-labeled ROS and Liperfluo-1-labeled lipid peroxides in CD4^+^ T cells, which were significantly reduced by Fer-1 intervention (Fig. 5A–B). Flow cytometry analysis revealed a higher proportion of CD4^+^ T cells in the PbA+Fer-1 group than in the PbA group (Fig. 5C), and this trend was consistent with the absolute cell counts (Fig. 5D). Further detection of cytokine secretion and subset polarization showed significantly increased proportions of IFN-γ^+^T-bet^+^ Th1 cells and TNF-α^+^CD4^+^ T cells in the PbA+Fer-1 group (Fig. 5E and F), indicating that Fer-1 promotes Th1-type immune responses. Treg cell assays showed that PbA infection significantly increased Treg proportions, which were further elevated by Fer-1 intervention (Fig. 5G), favoring the balancing of Th1 responses. Collectively, these results indicate that Fer-1 promotes Th1-type response polarization to enhance antimalarial cellular immunity by regulating oxidative stress in CD4^+^ T cells, while upregulating Treg levels to exert immunomodulatory effects and balance inflammatory levels in the host.

### 3.6 Fer-1 regulates macrophage oxidative stress and phenotypic polarization

**Fig. 6:**
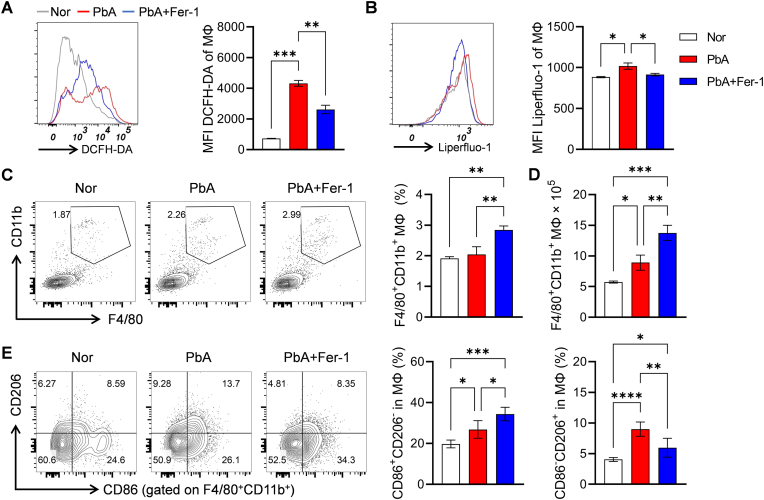
**Effects of Fer-1 on oxidative stress and phenotype of macrophages (MΦ) in PbA-infected mice**. **A.** Flow cytometry histograms and quantitative analysis MFI of ROS in MΦ labeled by DCFH-DA. **B.** Flow cytometry histograms and quantitative analysis MFI of lipid peroxidation in MΦ labeled by Liperfluo-1. **C.** Flow cytometry scatter plots and statistical bar charts of the proportion of F4/80^+^CD11b^+^ MΦ cells in spleen. **D.** Statistical bar charts of the absolute cell counts of F4/80^+^CD11b^+^ MΦ cells in spleen. **E.** Flow cytometry scatter plots and statistics of M1 (CD86^+^CD206^-^) and M2 (CD86^−^CD206^+^) MΦ subsets proportion. Data are presented as mean ± SD. Inter-group differences were analyzed by One-way ANOVA, *p < 0.05, **p < 0.01, ***p < 0.001, and ****p < 0.0001 considered statistically significant.

Splenic macrophages (MΦ) eliminate pathogens from the circulation through phagocytosis during malaria infection (Borges da Silva et al., 2015). Our results showed that PbA infection increased DCFH-DA-labeled ROS and Liperfluo-1-labeled lipid peroxides in splenic macrophages, which were significantly reduced by Fer-1 intervention (Fig. 6A–B). Flow cytometry analysis revealed that the proportion of F4/80^+^CD11b^+^ macrophages was significantly higher in the PbA + Fer-1 group compared to the PbA group (Fig. 6C), a trend that was consistently reflected in the absolute cell counts (Fig. 6D). Further detection of macrophage polarization showed that reducing systemic iron levels promoted MΦ polarization toward the CD86^+^CD206^-^ M1 phenotype, while decreasing the proportion of CD86^−^CD206^+^ M2 macrophages (Fig. 6E). Collectively, Fer-1 inhibits macrophage oxidative stress, regulates phenotypic polarization, and promotes M1-type macrophage polarization, which may enhance macrophage phagocytosis of iRBCs and result in lower parasitemia.

### 3.7 Fer-1 regulates cytokine secretion in splenocyte culture supernatants of PbA-infected mice

**Fig. 7:**
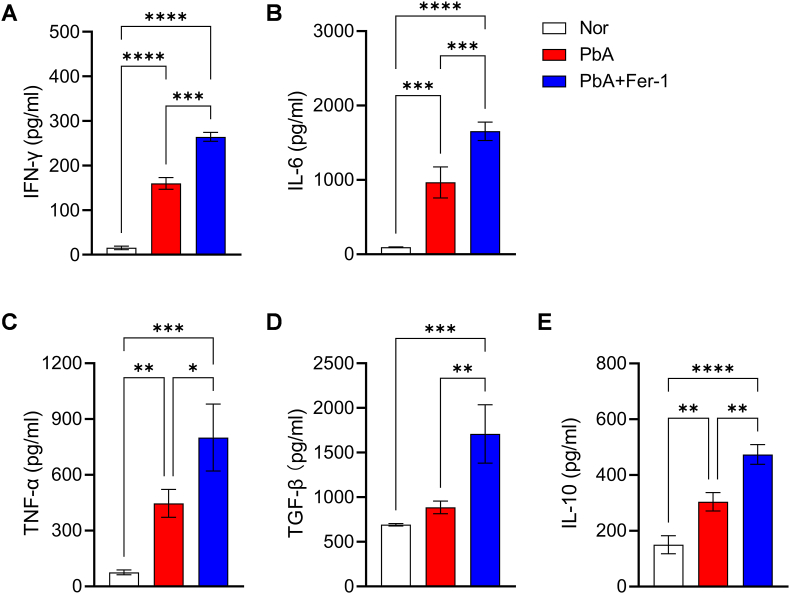
**Effect of Fer-1 on cytokine secretion in PbA-infected mice**. **A-E.** ELISA was used to detect the concentrations of IFN-γ, IL-6, TNF-α, TGF-β and IL-10. Data are presented as mean ± SD. Inter-group differences were analyzed by One-way ANOVA, *p < 0.05, **p < 0.01, ***p < 0.001 and ****p < 0.0001 considered statistically significant.

To investigate the effect of reduced systemic iron levels on host immune responses, in addition to analyzing the proportions and functions of splenic immune cells, we measured the secretion levels of key pro-inflammatory cytokines (IFN-γ, IL-6, and TNF-α) and anti-inflammatory cytokines (TGF-β and IL-10) in splenocyte culture supernatants. ELISA results showed that compared with the PbA group, IFN-γ and IL-6 secretion levels were significantly increased after Fer-1 administration (Fig. 7A and B), and TNF-α showed the same trend (Fig. 7C). Detection of anti-inflammatory cytokines revealed that both TGF-β and IL-10 secretion levels were significantly higher in the PbA + Fer-1 group than in the PbA group (Fig. 7D and E). These results suggest that reducing systemic iron levels promotes inflammatory responses while inducing anti-inflammatory effects to maintain host homeostasis and achieve balance.

### 3.8 Effects of Fer-1 intervention on cerebral ferroptosis in PbA-infected mice

**Fig. 8:**
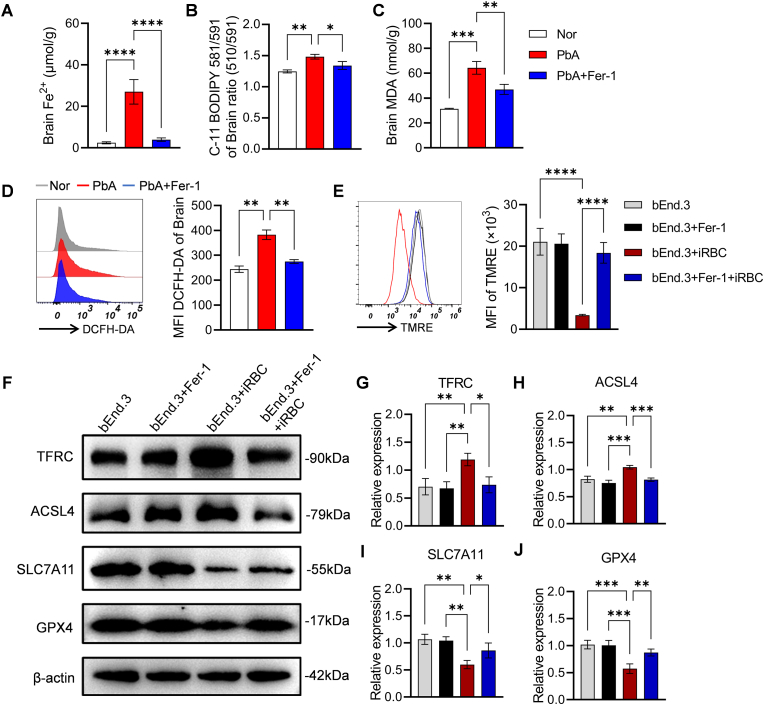
**Effects of Fer-1 on brain iron metabolism, oxidative stress, and related molecules**. **A.** Total iron content in brain tissue. **B.** Lipid peroxidation level detected by C11-BODIPY 581/591 (MFI) based on fluorescence quenching effect, and was calculated as the ratio of green fluorescence (510 nm) to red fluorescence (591 nm). **C.** Content of MDA in brain. **D.** Flow cytometry histograms and quantitative analysis MFI of ROS in brain labeled by DCFH-DA. **E.** Flow cytometry histograms and quantitative analysis MFI of mitochondrial membrane potential in bEnd.3 cells labeled by TMRE. **F.** Western blot analysis of ferroptosis-related proteins (TFRC, ACSL4, SLC7A11, GPX4) in bEnd.3 cells. **G-J.** Statistical analysis of relative expression level of TFRC, ACSL4, SLC7A11, and GPX4 protein. Data are presented as mean ± SD. Inter-group differences were analyzed by One-way ANOVA, with *p < 0.05, **p < 0.01, ***p < 0.001 and ****p < 0.0001 considered statistically significant.

Previous studies have indicated that disruption of the BBB accompanied by immune cell infiltration and neurotissue damage from hemorrhage represents the core pathogenic mechanism of CM (Nishanth and Schluter, 2019). Given that Fer-1 intervention enhanced the peripheral inflammatory immune response in PbA-infected mice, thereby contributing to control parasitemia, yet appears to contrast with its protective effect on the BBB, we hypothesize that Fer-1 may directly protect the brain endothelium and thus prolong the survival of infected mice. To test this hypothesis, both *in vivo* and *in vitro* experiments were conducted to investigate brain iron homeostasis. Our analyses revealed that PbA infection significantly increased brain iron content, lipid peroxidation levels, MDA content, and DCFH-DA-labeled ROS, all of which were reversed to normal levels by Fer-1 intervention (Fig. 8A–D). *In vitro* experiments using the murine brain microvascular endothelial cell line bEnd.3 demonstrated that Fer-1 treatment prevented the iRBC-induced decrease in intracellular MMP (Fig. 8E). Furthermore, iRBC stimulation significantly upregulated the expression of pro-ferroptotic proteins TFRC and ACSL4, while downregulating the expression of anti-ferroptotic proteins SLC7A11 and GPX4, indicating activation of endothelial ferroptosis (Fig. 8F–J). Notably, Fer-1 intervention reversed these changes and restored protein expression toward normal levels (Fig. 8F–J), whereas Fer-1 intervention restored these proteins’ expression (Fig. 8F–J-). This finding definitively confirms that Fer-1 effectively suppresses PbA infection-induced cerebral ferroptosis.

### 3.9 Effects of Fer-1 intervention on cerebral immune cell infiltration in PbA-infected mice

**Fig. 9:**
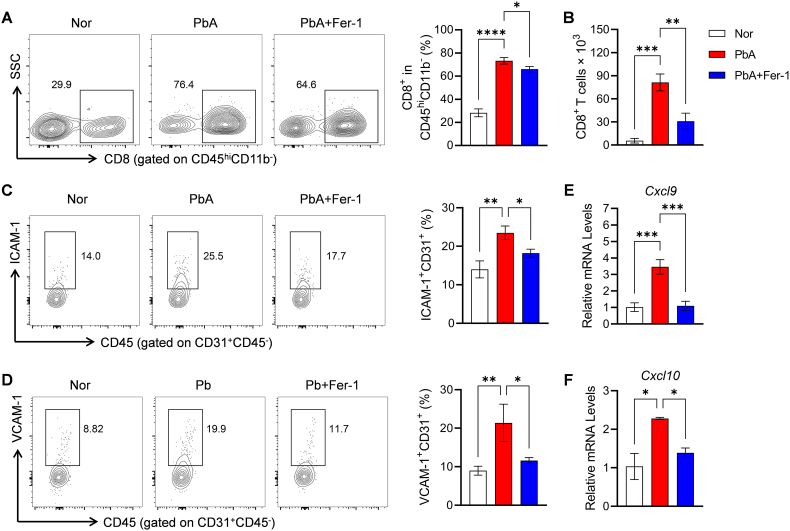
**Effects of Fer-1 on brain immune cells and related molecules in PbA-infected mice**. **A.** Flow cytometry scatter plots and statistics of CD8^+^ T cells proportion in brain CD45^+^CD11b^−^ lymphocyte. **B.** Statistics of CD8^+^ T cells absolute cell counts in brain CD45^+^CD11b^−^ lymphocyte. **C.** Flow cytometry scatter plots and statistics of ICAM-1^+^ proportion in brain CD31^+^CD45^−^ endothelial cells. **D.** Flow cytometry scatter plots and statistics of VCAM-1^+^ proportion in brain CD31^+^CD45^−^ endothelial cells. **E.** Detection of relative mRNA level of *Cxcl9.***F.** Detection of relative mRNA level of *Cxcl10*. Data are presented as mean ± SD. Inter-group differences were analyzed by One-way ANOVA, with *p < 0.05, **p < 0.01, ***p < 0.001 and ****p < 0.0001 considered statistically significant.

Current research suggests that cerebral infiltration of CD8^+^ T cells is a key pathogenic factor mediating ECM (Howland et al., 2015). Flow cytometry analysis of brain CD8^+^ T cells showed that PbA infection significantly increased both the proportion and absolute number of CD8^+^ T cells in the brain compared to the control group. In contrast, Fer-1 treatment not only reduced the proportion of CD8^+^ T cells in the brain but also significantly decreased their absolute number, confirming the inhibitory effect of Fer-1 on the brain infiltration of CD8^+^ T cells (Fig. 9A and B). Additionally, abnormal expression of ICAM-1^+^ and VCAM-1^+^ on brain microvascular endothelial cells is critical for immune cell adhesion and vascular obstruction. We found that PbA infection significantly increased the proportion of ICAM-1^+^/VCAM-1^+^ endothelial cells, while Fer-1 treatment downregulated these adhesion molecules (Fig. 9C and D). Mechanistic studies further revealed that PbA infection markedly upregulated the mRNA expression of brain chemokines *Cxcl9* and *Cxcl10*, which was significantly suppressed by Fer-1 treatment (Fig. 9E and F), consistent with the reduced cerebral CD8^+^ T cell infiltration phenotype.

## 4 Discussion

The escalating problem of drug resistance and the lag in the development of new antiparasitic drugs pose a serious threat to malaria treatment (Nishanth and Schluter, 2019), making it crucial to explore more effective therapeutic approaches. Iron homeostasis is closely associated with malaria pathogenesis (Adegboro and Afolabi, 2024), yet how iron homeostasis regulates immune responses in the spleen and brain during the development of CM remains poorly understood. This study found that after PbA infection, serum iron levels in mice significantly increased, along with remarkable elevations in ROS and MDA levels in the spleen and brain, exacerbating lipid peroxidation, which is consistent with previous reports (Das et al., 1991; Reis et al., 2010; Liang et al., 2022). Studies have shown that host cells influence the role of iron in regulating malaria immune responses through transferrin receptor 1 (TFR1), affecting both pathogen control and host adaptive immunity (Wideman et al., 2023). Our results also demonstrated increased TFR1 expression in CM. Additionally, we focused on key regulators influencing iron homeostasis through different pathways: ACSL4, SLC7A11, and GPX4. Research indicates that ACSL4, as a key lipoxygenase, shows a positive correlation between its expression and the severity of lipid metabolic imbalance (Guo, 2022). While SLC7A11 and GPX4 constitute the main antioxidant defense mechanism against oxidative stress caused by iron homeostasis imbalance, with their expressions negatively correlated with the severity of iron imbalance (Chen et al., 2021; Liu et al., 2024b). Our findings revealed that after PbA infection, ACSL4 expression increased in splenocytes, while SLC7A11 and GPX4 expressions significantly decreased, indicating that malaria infection leads to iron homeostasis imbalance and elevated lipid peroxidation in the host.

Recent studies have found that Fer-1 can significantly block Lipocalin-2-mediated BBB disruption by inhibiting ferroptosis in cerebral vascular endothelial cells (Liu et al., 2024a). In view of this, we further explored the therapeutic potential of iron homeostasis regulation in CM. Results showed that Fer-1 significantly reduced splenic ROS levels and lipid peroxidation, effectively reversing iron metabolic disorders in ECM mice. Phenotypic observations demonstrated that Fer-1 treatment led to a marked decrease in parasitemia and a significant extension of survival, further confirming the decisive impact of iron homeostasis regulation on CM progression. Notably, existing studies have shown controversies regarding the antimalarial effects of iron intervention: early research indicated that nutritional iron deficiency could reduce parasitemia by inducing suicidal death of red blood cells and improve survival in infected mice (Koka et al., 2007), while recent studies on *Plasmodium chabaudi* found that iron uptake restriction instead increased parasitemia and impaired immune responses (Wideman et al., 2023). This contradiction may be attributed to the pathological heterogeneity of different murine malaria models. Combining the results of this study with existing controversies, we argue that in addition to the role of red blood cells themselves in iron metabolism, more attention should be focused on the dynamic changes in the host's antimalarial immune response following improvement of iron homeostasis.

As a core hub connecting innate and adaptive immunity, DCs mature and migrate to the spleen and lymph nodes upon recognition of foreign antigens. They present antigen peptides via MHC class II molecules while upregulating costimulatory molecules such as CD80 and CD86, driving the differentiation of naïve T cells into effector T cells (Yap et al., 2019). Previous studies have shown that iron overload in the tumor microenvironment can induce iron-dependent death of DCs, significantly impairing their antigen-presenting capacity and thereby promoting tumor progression (Wiernicki et al., 2022). Our results demonstrate that Fer-1 intervention, by correcting iron metabolic imbalance in CM mice, significantly promotes DC activation, manifested by marked upregulation of MHC class II molecules and costimulatory molecules CD80 and CD86, thus facilitating adaptive immune responses.

In the malaria immune regulatory network, CD4^+^ T cells and macrophages constitute key roles: IFN-γ secreted by Th1 cells plays a central role in controlling acute infection (Inoue et al., 2013; Gbedande et al., 2020), while macrophages eliminate *Plasmodium* through phagocytic killing mechanisms (Borges da Silva et al., 2015; Dobbs et al., 2020; Kumar et al., 2020). Studies have reported that iron chelators effectively reduce intracellular ROS levels in T cells and decrease DNA oxidative damage under iron overload conditions (Shaw et al., 2017). This study further confirms that Fer-1 treatment not only significantly reduces ROS and lipid peroxidation levels in CD4^+^ T cells and macrophages but also enhances early antimalarial immune effects and effectively controls parasitemia by promoting Th1 cell polarization and M1-type transformation of macrophages. Notably, Fer-1 intervention simultaneously upregulates the proportion of Treg cells, forming a dynamic balance of "pro-inflammatory-anti-inflammatory". This result is consistent with reports that iron chelators promote Treg cell proliferation in autoimmune diseases (Lai et al., 2022), suggesting that iron homeostasis regulation exerts a dual regulatory role in limiting excessive inflammatory responses and alleviating CM neuropathological damage.

During malaria infection, the dynamic balance between pro-inflammatory and regulatory immunity is a critical factor determining host infection clearance and prolonged survival. When this balance is disrupted, excessively activated pro-inflammatory responses trigger severe malaria-related pathological damage, serving as a major contributor to high mortality (Dobbs et al., 2020). During the blood infection stage, pro-inflammatory cytokines such as IL-1β, IL-6, IL-8, IL-12, IFN-γ, and TNF-α released by the host immune system play a decisive role in antimalarial immunity by regulating immune cell activation and pathogen clearance (Mitchell et al., 2005; Vigario et al., 2007). Concurrently, the body synchronously produces regulatory cytokines like TGF-β and IL-10 to maintain immune homeostasis by inhibiting excessive inflammatory responses (Popa and Popa, 2021; Abosalif et al., 2023). Our results demonstrate that Fer-1 intervention significantly enhances the secretion of pro-inflammatory cytokines including IFN-γ, TNF-α, and IL-6, effectively boosting antimalarial immune effects and reducing parasitemia levels. Meanwhile, Fer-1 coordinately promotes the release of TGF-β and IL-10, precisely regulating the intensity of immune responses to avoid immunopathological damage and maintain immune homeostasis in the body.

BBB disruption contributes to neural injury, characterized by the breakdown of tight junctions and impaired endothelial cell function, which facilitate immune cell transmigration across the BBB into the central nervous system (Bernard-Valnet et al., 2016; Eeka and Phanithi, 2018). A recent study has demonstrated that ferroptosis is involved in the pathogenesis of neuronal damage in cerebral malaria, with CD8^+^ T cells playing a critical role in inducing neuronal ferroptosis (Liang et al., 2022). Meanwhile, evidence indicates that during severe malaria infection, heme-derived nanoparticles in the circulation deliver excessive iron to brain endothelial cells, triggering intense cerebral oxidative stress and inflammatory responses, ultimately leading to compromised BBB integrity (Nguyen et al., 2023). In this study, we found that Fer-1 intervention effectively restored cerebral iron homeostasis, prevented infection-induced ferroptosis in brain endothelial cells, and significantly improved BBB function. These findings suggest that Fer-1 treatment may protect against the development of ECM by exerting dual protective effects on both brain endothelial cells and the central nervous system.

It is currently widely accepted that the pathogenesis of ECM in mice is primarily mediated by CD8^+^ T cells migrating to the brain, which directly secrete perforin and granzyme B (Potter et al., 2006; Haque et al., 2011). Cerebrovascular endothelial cells highly express ICAM-1 and VCAM-1, which may serve as antigen-specific targets recognized by CD8^+^ T cells, thereby inducing BBB dysfunction, vascular leakage, and neuronal death (Howland et al., 2013; Swanson et al., 2016). The cerebral migration of CD8^+^ T cells depend on chemokines CXCL9 and CXCL10 abundantly secreted by cerebrovascular endothelial cells after infection (Campanella et al., 2008; Sorensen et al., 2018). Our results showed that Fer-1 intervention significantly reduced the proportion of cerebral CD8^+^ T cells, simultaneously downregulated the transcriptional levels of *Cxcl9* and *Cxcl10*, and decreased the number of endothelial cells expressing ICAM-1 and VCAM-1. *In vitro* experiments demonstrated that Fer-1 effectively blocked iRBC-induced endothelial ferroptosis. These findings indicate that Fer-1 protects mice from ECM pathological damage by inhibiting brain endothelial ferroptosis, reducing chemokine and adhesion molecule expression, and thus preventing abnormal CD8^+^ T cell infiltration.

In conclusion, this study confirms that ferroptosis is involved in the pathological process of CM. The ferroptosis inhibitor Fer-1 regulates iron homeostasis, reshapes immune responses, and protects the blood-brain barrier, providing a novel strategy for antimalarial therapy.

## CRediT authorship contribution statement

**Shijie Yao:** Writing – original draft, Methodology, Formal analysis, Data curation. **Xiaoliang Zhou:** Writing – original draft, Methodology, Formal analysis, Data curation. **Ting Liao:** Methodology, Formal analysis. **Chao Yao:** Data curation, Methodology. **Mengna Sun:** Methodology, Formal analysis. **Haojie Gou:** Methodology, Formal analysis. **Xueyuan Hu:** Methodology, Formal analysis. **Junyu Liu:** Methodology, Formal analysis. **Li Zheng:** Writing – review & editing, Writing – original draft, Conceptualization. **Yan Zhao:** Writing – review & editing, Writing – original draft, Conceptualization. **Yaming Cao:** Writing – review & editing, Funding acquisition, Conceptualization.

## Ethical approval

All procedures were approved by university internal review committees, including the Education and Ethics Committees.

## Conflict of interest

The research was conducted without commercial or financial relationships for any of the authors. Contributions from all authors were reviewed and approved before the article was submitted. The authors declare that they have no competing interests.
